# Targeting of CD133+ Cancer Stem Cells by Mesenchymal Stem Cell Expressing TRAIL Reveals a Prospective Role of Apoptotic Gene Regulation in Non-Small Cell Lung Cancer

**DOI:** 10.3390/cancers11091261

**Published:** 2019-08-28

**Authors:** Kamal Shaik Fakiruddin, Moon Nian Lim, Norshariza Nordin, Rozita Rosli, Zubaidah Zakaria, Syahril Abdullah

**Affiliations:** 1 UPM-MAKNA Cancer Research Laboratory, Institute of Bioscience, Universiti Putra Malaysia, Selangor 43400, Malaysia; 2 Haematology Unit, Cancer Research Centre, Institute for Medical Research (IMR), National Institutes of Health (NIH), Ministry of Health Malaysia, Shah Alam 40170, Malaysia; 3 Medical Genetics Laboratory, Department of Biomedical Sciences, Faculty of Medicine & Health Sciences, Universiti Putra Malaysia, Selangor 43400, Malaysia; 4 Genetics and Regenerative Medicine Research Centre, Faculty of Medicine & Health Sciences, Universiti Putra Malaysia, Selangor 43400, Malaysia

**Keywords:** mesenchymal stem cell, TRAIL, cytotherapy, apoptosis, cancer stem cells, non-small cell lung cancer

## Abstract

Mesenchymal stem cells (MSCs) are emerging as vehicles for anti-tumor cytotherapy; however, investigation on its efficacy to target a specific cancer stem cell (CSC) population in non-small cell lung cancer (NSCLC) is lacking. Using assays to evaluate cell proliferation, apoptosis, and gene expression, we investigated the efficacy of MSCs expressing tumour necrosis factor (TNF)-related apoptosis inducing ligand (MSC-TRAIL) to target and destroy CD133+ (prominin-1 positive) NSCLC-derived CSCs. Characterization of TRAIL death receptor 5 (DR5) revealed that it was highly expressed in the CD133+ CSCs of both H460 and H2170 cell lines. The human MSC-TRAIL generated in the study maintained its multipotent characteristics, and caused significant tumor cell inhibition in NSCLC-derived CSCs in a co-culture. The MSC-TRAIL induced an increase in annexin V expression, an indicator of apoptosis in H460 and H2170 derived CD133+ CSCs. Through investigation of mitochondria membrane potential, we found that MSC-TRAIL was capable of inducing intrinsic apoptosis to the CSCs. Using pathway-specific gene expression profiling, we uncovered candidate genes such as *NFKB1*, *BAG3*, *MCL1*, *GADD45A*, and *HRK* in CD133+ CSCs, which, if targeted, might increase the sensitivity of NSCLC to MSC-TRAIL-mediated inhibition. As such, our findings add credibility to the utilization of MSC-TRAIL for the treatment of NSCLC through targeting of CD133+ CSCs.

## 1. Introduction

Lung cancer is the most frequently diagnosed cancer worldwide, accounting for more than 1.7 million (18.4%) of total cancer deaths in 2018 [1]. Non-small cell lung cancer (NSCLC) represents 80% of all lung cancer cases, followed by small-cell lung cancer [2]. Classical chemotherapies and platinum drugs (e.g., cisplatin or carboplatin), combined with third-generation compounds such as vinorelbine and gemcitabine, may improve NSCLC patient overall survival. However, these treatments would eventually fail due to acquired chemoresistance and microscopic tumor dissemination [3]. The anti-tumor efficacy of engineered mesenchymal stem cells (MSCs), expressing cytokines such as interleukins [4,5] and interferons [6], or oncolytic viruses [7,8], paved the way toward an alternative approach of using engineered MSCs as cytotherapy against different tumors, including NSCLC. However, safety concerns, such as off-tumor toxicity induced by the cytokines (e.g., hematological toxicities, autoimmune, and hepatotoxicity) [9,10] and the potential of unspecific targeting or reversion of oncolytic viruses into wild type [11], hampered its application in the clinic.

In contrast, TRAIL (tumour necrosis factor-related apoptosis inducing ligand) was reported to be highly effective in targeting and destroying a variety of tumors, including colorectal cancer [12], glioblastoma [13], and NSCLC [14], without any toxicities. This is due to the high TRAIL cognate death receptors 4 and 5 (DR4 and DR5) expression in most tumors, but not in normal cells [15]. TRAIL can exist either as a soluble ligand or transmembrane protein, and induces apoptosis through binding to its specific cognate receptors. Binding of TRAIL to its receptors initiates a signaling cascade that activates extrinsic apoptosis through *CASP8/10* and intrinsic apoptosis through cytochrome C release from the mitochondria. However, due to its short half-life and likelihood to be eliminated through renal filtration, TRAIL needs a delivery system to be effective [16]. To date, several recombinant variants of human TRAIL were developed to increase its tumor-killing potential [17,18]. For example, the efficacy of TRAIL through the paracrine effect was enhanced by the addition of an immunoglobulin chain into the structure of TRAIL [19]. The addition of tags such as an isoleucine zipper resulted in the stabilization of TRAIL trimmers compared to the native TRAIL [20]. Although many recombinant human TRAILs were found to be safe and effective, some of them did not have sufficient therapeutic effect for clinical trial. This is partly due to the intrinsic and acquired resistance of most tumors to the TRAIL treatment [21]

Mesenchymal stem cells, also known as mesenchymal stromal cells or MSCs, are adult multipotent stem cells that can be derived from several sources such as adipose tissue [22], peripheral blood [23], umbilical cord [24], and bone marrow [25]. MSCs hold great potential as cytotherapy compared to other stem cells due to their high expansion capacity, ease of isolation, being immune-privileged due to lack of major histocompatibility complex (MHC) class II, and ability to exert paracrine activity at the target site [26]. Although the immune-privileged property of MSCs is contentious [27,28,29], the remarkable benefits of MSCs as an immune modulator in patients experiencing graft versus host disease may outweigh their side effects [30,31,32,33]. These cells, initially believed to have a restricted differentiation capacity to mesodermal lineage and applied only for regenerative medicine, are now proven by numerous reports to be more robust [34,35,36,37,38]. Several reports showed the capacity of engineered MSCs expressing TRAIL (MSC-TRAIL) homing to the tumor microenvironment and inducing significant tumor regression [39,40,41]. The effect of MSC-TRAIL destroying tumors was well described in pre-clinical models of glioblastoma [42], pancreatic tumor [43,44], breast cancer [45,46,47,48], and prostate cancer [49]. However, very few studies reported the anti-tumor efficacy of MSC-TRAIL in lung cancer, and its ability to inhibit cancer stem cells (CSCs) derived from NSCLC. One study reported the capacity of MSC-TRAIL to inhibit CSCs derived from a side population of NSCLC [50]; however, its efficacy in targeting and destroying other CSC populations in NSCLC is not well documented.

Cancer stem cells (CSCs) are a small population of tumors, known to be the cause of chemoresistance and tumor relapse [51]. Several markers of CSCs were identified in lung cancer such as homing cell adhesion molecule (CD44) [52], aldehyde dehydrogenase (ALDH) [53], CD326 [54], and CD133 [55]. Recently, novel approaches were developed using nanoparticles [56,57,58,59,60] or antibody-conjugated nanoparticles [61,62] to target these CSCs. Although these methods may seem promising, safety issues such as specificity, off-target accumulation, cellular toxicity [63,64], and impact on the environment [65] are some of the concerns that may hamper their progress to clinical application. MSCs on the other hand may serve as an alternative for a safer approach considering that these cells are widely used for the treatment of several degenerative diseases with very few side effects [66,67,68].

MSCs were utilized as a factory for drug production [69], and a delivery system for different biological agents including pro-drug converting enzymes [70,71], anti-tumor cytokines [72,73,74], and oncolytic viruses [75,76]. Although, several cancer models including glioblastoma [77], ovarian tumor [78], and melanoma [79] were tested for the anti-tumor efficacy of MSC-TRAIL, studies that show the efficacy of MSC-TRAIL to target cancer stem cells (CSCs) from NSCLC are still insufficiently reported. Therefore, to understand and later develop such a strategy, we evaluated the efficacy of MSCs expressing TRAIL (MSC-TRAIL) to target and kill CD133+ CSCs in NSCLC using several assays related to cell proliferation and the apoptosis of both the intrinsic and extrinsic pathways. We furthered our investigations by identifying genes involved in TRAIL resistance using pathway-specific reverse transcriptase (RT^2^) profiler PCR arrays. We believe that these genes may be the key regulators that control the sensitivity of CSCs to TRAIL and MSC-TRAIL-mediated inhibition.

## 2. Results

### 2.1. Characterization of MSC-TRAIL

**Figure 1:**
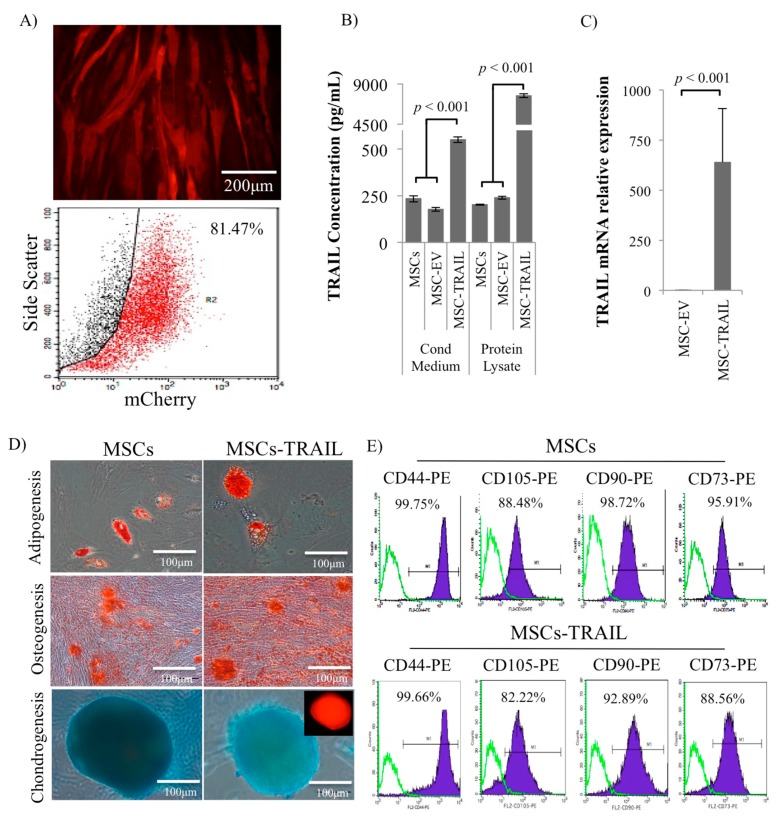
Verified mesenchymal stem cell expressing tumour necrosis factor (TNF)-related apoptosis inducing ligand (MSC-TRAIL) maintained its multipotent characteristics. (**A**) Representative microscopy images and flow cytometry analysis of mCherry in MSCs transduced with TRAIL-encoding lentivirus (MSC-TRAIL). (**B**) Analysis of TRAIL concentration by ELISA in both conditioned medium and protein lysate of MSCs (MSCs, MSC-EV (empty vector), and MSC-TRAIL). (**C**) Messenger RNA (mRNA) expression of TRAIL in transduced MSCs by qRT-PCR. (**D**) MSC-TRAIL and MSC differentiation: adipogenesis (Oil-Red O), osteogenesis (Alizarin Red), and chondrogenesis (Alcian Blue)/fluorescence. (**E**) MSC surface markers, cluster of differentiation (CD44, CD90, CD105, and CD73) analysis from MSC-TRAIL and MSCs by flow cytometry (*p* < 0.001; *t*-test).

MSCs were transduced with lentivirus encoded either with TRAIL or without TRAIL (empty vector (EV)), both tagged with mCherry. The transduction unit, TU/mL, was calculated by infecting Lenti-X 293T cells with TRAIL-encoded lentivirus, and the multiplicity of infection (MOI) in MSCs that yielded the highest transduction efficiency was determined based on the calculated TU/mL. MOI 20 was used for TRAIL-encoded lentivirus in subsequent experiments that produced 81.47 ± 0.3% mCherry-positive gated for MSC-TRAIL at 72 h (Figure 1A). Analysis of the transduction efficiency by flow cytometry was performed at 72 h due to the highest intensity of mCherry/red fluorescence protein (RFP)-positive cells detected in transduced cells. Significantly higher (*p* < 0.001) TRAIL protein was detected in the protein lysate and conditioned medium of MSCs transduced with the TRAIL-encoded lentivirus (MSC-TRAIL) as compared to the empty vector (MSC-EV) and wild-type MSCs using ELISA (Figure 1B). Substantially higher TRAIL messenger RNA (mRNA) was noted in MSC-TRAIL as compared to MSC-EV as analyzed by quantitative RT-PCR (Figure 1C).

MSC-TRAIL was induced to mesodermal lineage differentiation (adipogenesis, osteogenesis, and chondrogenesis) and histologically stained (Oil-Red O, Alizarin Red, and Alcian Blue) for verification. Our findings indicated that the transduced MSCs (MSC-TRAIL) maintained their multipotent characteristics (Figure 1D). MSC-TRAIL was positive for all three stainings, indicating adipogenesis, osteogenesis, and chondrogenesis differentiation (Figure 1D). MSC-TRAIL also maintained its MSC surface markers expression (CD44, CD90, CD105, and CD73) as analyzed by flow cytometry (Figure 1E).

### 2.2. Targeting of NSCLC Cell Lines by Recombinant Human (rh) TRAIL (rhTRAIL) and Its TRAIL Receptors Expression

**Figure 2:**
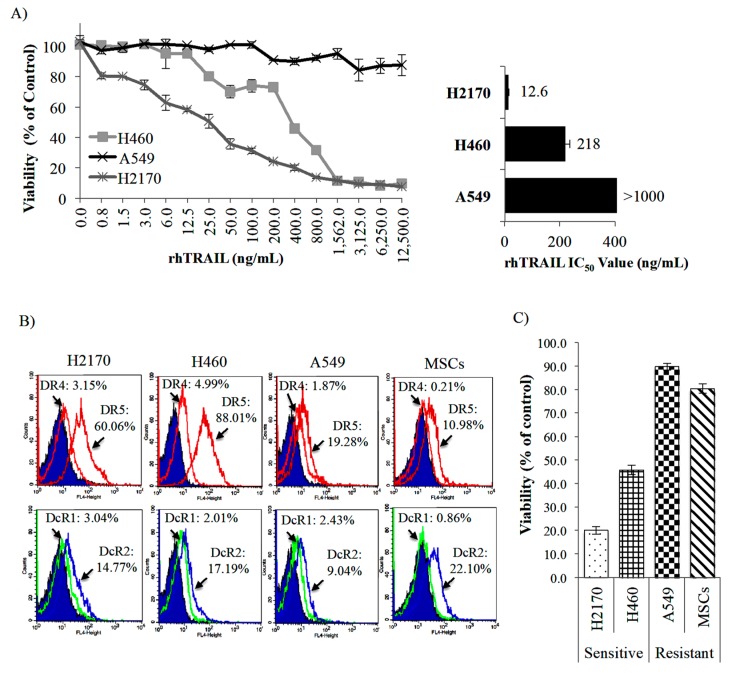
TRAIL sensitivity by in vitro characterization. (**A**) The half maximal inhibitory concentration (IC_50_) value was determined in non-small cell lung cancer (NSCLC) cell lines (H460, H2170 and A549) by MTS assay after 48 h of rhTRAIL treatment at different concentrations. (**B**) Analysis of TRAIL receptors expression [agonist receptors (DR4 and DR5) and decoy receptors (DcR1 and DcR2)] in MSCs and NSCLC cell lines by flow cytometry. (**C**) Sensitivity of NSCLC cell lines and MSCs evaluated by MTS assay after rhTRAIL (500 ng/mL) treatment.

The MTS [3-(4,5-dimethylthiazol-2-yl)-2H-tetrazolium, inner salt] assay (proliferation assay) performed in the NSCLC cell lines (H460, H2170, and A549) in the presence of different rhTRAIL concentrations showed that each of these cell lines responded differently to the rhTRAIL treatment (Figure 2A, left panel). The H2170 cell line was highly responsive to rhTRAIL, followed by the H460 cell line with half maximal inhibitory concentration (IC_50_) values of 12.6 and 218 ng/mL, respectively (Figure 2A, right panel). However, the A549 was unresponsive to the rhTRAIL treatment (Figure 2A, right panel). Sensitive cell lines (H2170 and H460) expressed the highest DR5 receptor expression (60.06% and 88.01%), followed by resistant cell lines (A549 and MSCs) with only 19.28% and 10.98%, respectively (Figure 2B). Moreover, both H2170 and H460 cell lines expressed slightly higher DR4 receptor expression (3.15% and 4.99%, respectively) than A549 (1.87%) and MSCs (0.21%). The expression of TRAIL decoy receptors (DcR1 and DcR2) was consistently low and almost similar across all the NSCLC cell lines (Figure 2B). Based on these results, we suggest that the sensitivity of these NSCLC cell lines to rhTRAIL might be correlated to the expression of TRAIL cognate receptor DR5.

### 2.3. Isolation of CD133+ from NSCLC Cell Lines and Its DR5 Receptor Expression

**Figure 3:**
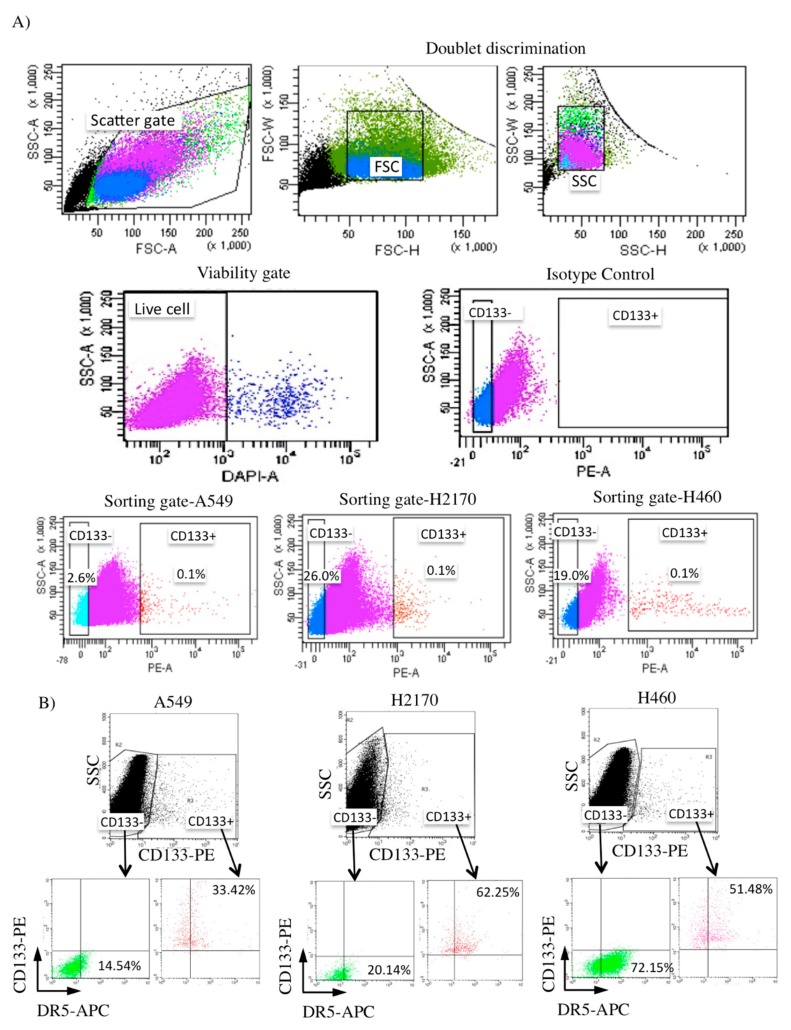
Flow cytometry analysis of CD133+ NSCLC-derived cancer stem cells (CSCs) and their TRAIL receptor expression. (**A**) Cell debris and doublets were discriminated, and dead cells were excluded [(DAPI/4′,6-diamidino-2-phenylindole) negative; shown by the first four panels], followed by isolation of the CD133+ population in NSCLC cell lines (A549, H2170, and H460) in the lower panels. (**B**) Both CD133+ CSCs of H2170 and H460 expressed a high level of TRAIL cognate receptor (DR5), consistent with their sensitivity to TRAIL.

To identify the population of CSCs, we investigated the expression of CD133+ in these NSCLC cell lines (A549, H2170, and H460). The expression of CD133+ in all of the NSCLC cell lines was low (~0.1% expression), consistent with a previous report that identified CSCs in lung cancer samples (Figure 3A) [80]. The CD133+ (CSC) and CD133− (non-CSC) populations were isolated and further purified by culturing and sorting for a total of three independent experiments. Analysis of the TRAIL cognate receptor expression (DR5) revealed that it is considerably higher in the CD133+ CSCs of both H2170 (62.25%) and H460 (51.48%) than A549 (33.42%), consistent with its sensitivity to rhTRAIL treatment (Figure 3B).

### 2.4. Greater Sphere Formation and Clonogenicity in CD133+ NSCLC-Derived CSCs

**Figure 4:**
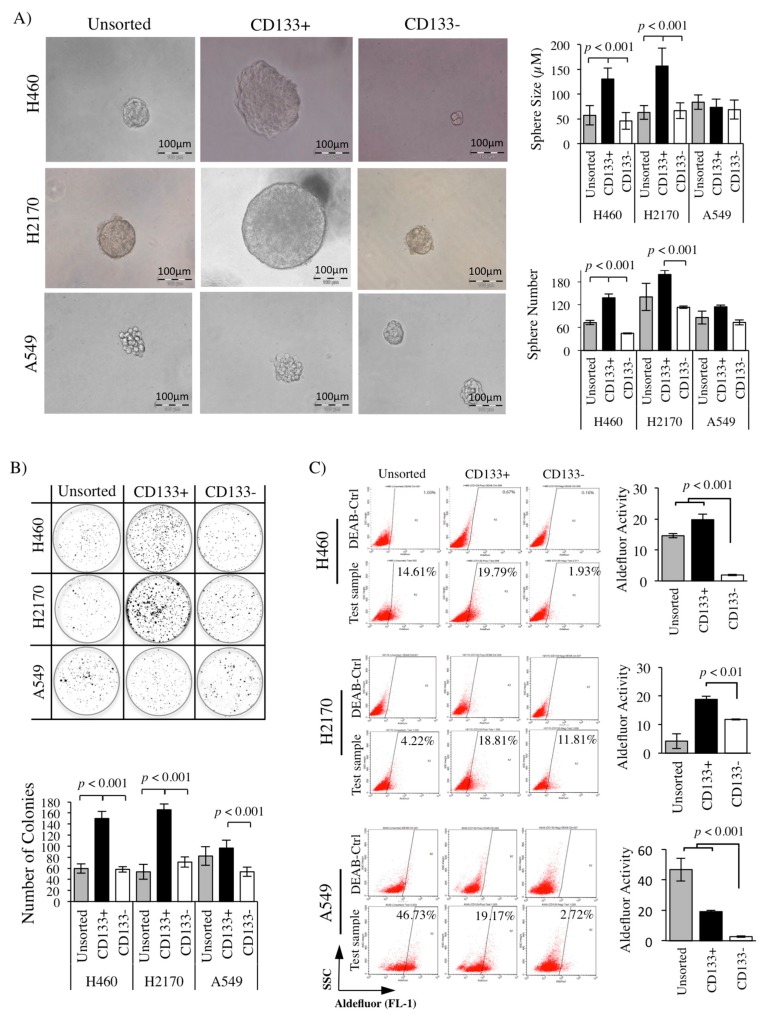
Isolated CD133+ populations exhibit the characteristics of CSCs. (**A**) Sphere formation of CD133+ population derived from NSCLC cell lines in anchorage-independent culture. Substantially bigger and a higher number of spheres in the CD133+ CSCs as compared to control (CD133− and unsorted) in both H460 and H2170 cell lines. (**B**) A significant number of colonies was noted in the CD133+ CSCs as compared to CD133− (non-CSCs) population in all of the NSCLC cell lines. (**C**) Aldefluor assay indicating high ALDH activity in the CD133+ NSCLC-derived CSCs (*p* < 0.001; *t*-test).

Sphere formation and clonogenicity analyses were performed to verify the CSCs characteristics of the CD133+ population derived from NSCLC cell lines (H460, H2170, and A549). Significantly bigger spheres (*p* < 0.001) were noted in H460- and H2170-derived CD133+ CSCs, reaching an average diameter of 130.47 ± 22.2 and 156.66 ± 36.48 μM, respectively, compared to the CD133− (45.71 ± 17.11 and 66.45 ± 15.83 μM) and unsorted (56.99 ± 19.28 and 62.88 ± 13.84 μM) cell lines (Figure 4A). In addition, a higher number of spheres (*p* < 0.001) was detected in the CD133+ population derived from H460 and H2170 with an average sphere number of 138 ± 9.5 and 199 ± 11.3, compared to a lower number of spheres in the CD133− (45.0 ± 1.4 and 113 ± 2.8) population (Figure 4A). However, there was no significant difference in the number and size of spheres in the A549-derived CD133+ population as compared to the controls (CD133− and unsorted).

CD133+ NSCLC-derived CSCs and controls (CD133− and unsorted) were seeded at a very low density (1000 cells in 2 mL) in a six-well plate. After 14 days, formed colonies were stained and manually counted. The H460-, H2170-, and A549-derived CD133+ CSC population presented a significantly higher (*p* < 0.001) number of colonies with 150.0 ± 12.9, 165.8 ± 10.4, and 96.6 ± 3.8 than CD133− with only 58.14 ± 4.9, 71.2 ± 9.0, and 53.6 ± 8.2 colonies detected, respectively (Figure 4B).

### 2.5. High ALDH Activity Detected in the CD133+ NSCLC-Derived CSCs

Reports showed that high ALDH activity is an important indicator for chemoresistance [81]. As CSCs are known to be the culprit that contributes toward chemoresistance in most tumors, it is expected that CSCs have a high ALDH activity compared to the non-CSCs. The activity of ALDH can be detected using a specific fluorescence stain, such as the Aldefluor assay (STEMCELL Technologies, Vancouver, Canada), and analyzed using flow cytometry. Our analysis revealed that the ALDH activity was significantly higher in the CD133+ CSC population derived from H460, H2170, and A549 with 19.8 ± 1.8%, 18.8 ± 1.0%, and 19.2 ± 0.7% expression levels detected, compared to the CD133− (non-CSCs) with only 1.9 ± 0.2%, 11.8 ± 0.2%, and 2.7 ± 0.6% (Figure 4C).

### 2.6. Inhibition of CD133+ CSC Proliferation by MSC-TRAIL

**Figure 5:**
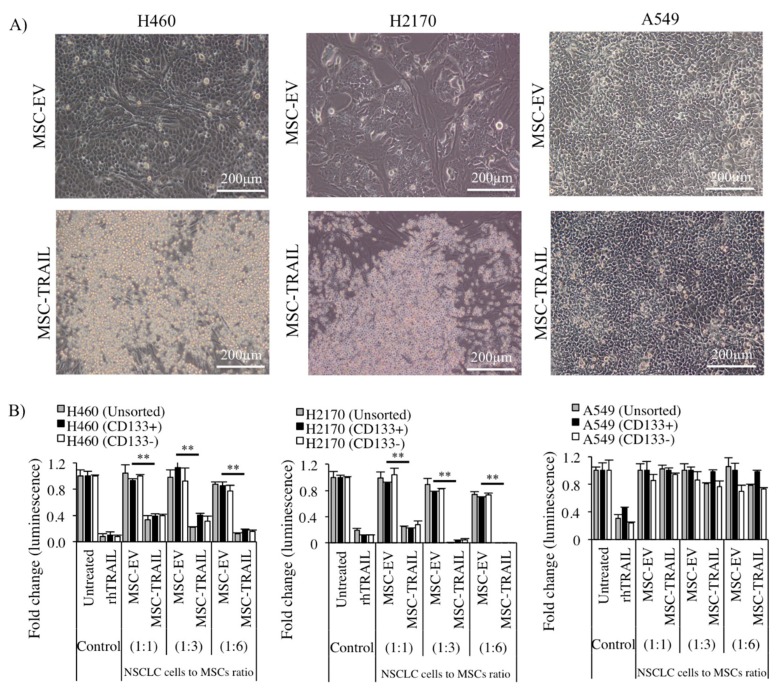
Inhibition of CD133+ CSCs by MSC-TRAIL. (**A**) Representative images taken from phase-contrast microscopy of CD133+ NSCLC (H460, H2170, and A549)-derived CSCs co-cultured with either MSC-TRAIL or MSC-EV. Microscopic examination revealed the presence of dead cells/apoptotic bodies in the CD133+ CSCs derived from H460 and H2170 cell lines treated with MSC-TRAIL. (**B**) The viability of sorted (CD133+ and CD133−) and unsorted NSCLC cell lines (H460, H2170, and A549) co-cultured with either MSC-EV or MSC-TRAIL at different ratios. The rhTRAIL treatment (IC_50_ value) was used as the positive control (** *p* < 0.001; *t*-test).

The sorted (CD133+ and CD133−) and unsorted NSCLC cell lines were co-cultured with MSCs (MSC-TRAIL or MSC-EV) at different NSCLC-cell-to-MSC ratios (1:1, 1:3, and 1:6) in a 96-well tissue culture plate for 72 h, and rhTRAIL was used as a positive control for the treatment. Under phase-contrast microscopy, the formation of apoptotic bodies and dead cells was clearly seen in both H460- and H2170-derived CD133+ cells cultured with MSC-TRAIL when compared to the MSC-EV (cultured at a 1:1 NSCLC-cell-to-MSC ratio), depicted in Figure 5A. However, no apoptotic body was detected in the A549 cell line. Significant inhibition (*p* < 0.001) of CD133+ CSC viability from both H460 and H2170 was detected when these cells were co-cultured with MSC-TRAIL starting from a ratio of 1:1, and the cytotoxic effect of MSC-TRAIL was more evident as the ratio increased (Figure 5B). However, no inhibition was detected in A549, suggesting that the cell line was resistant to the MSC-TRAIL treatment. The results also indicate that MSC-EV did not cause NSCLC cell death, signifying a specific action of secreted TRAIL and not MSCs (Figure 5B).

### 2.7. MSC-TRAIL Induced Annexin V Expression in CD133+ CSCs

**Figure 6:**
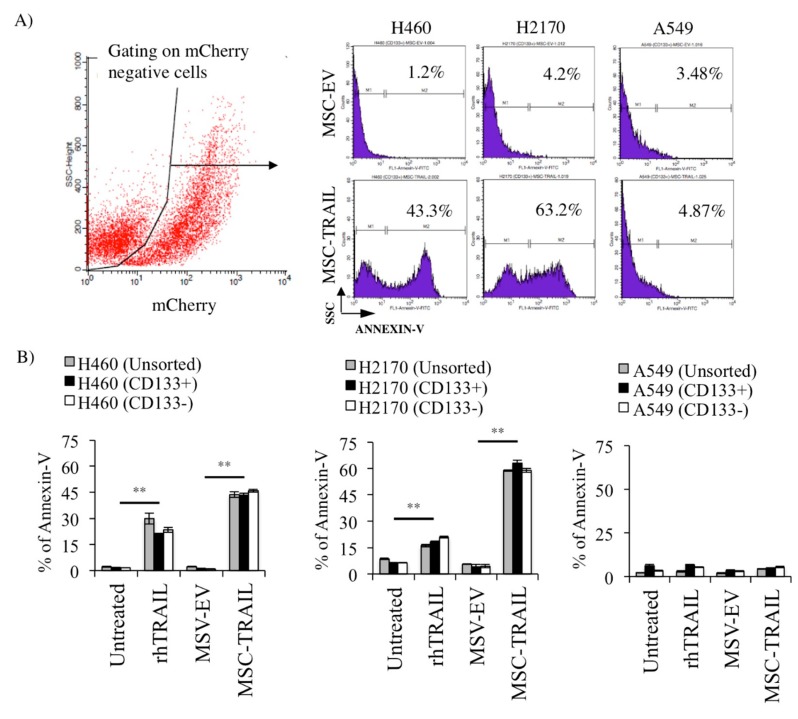

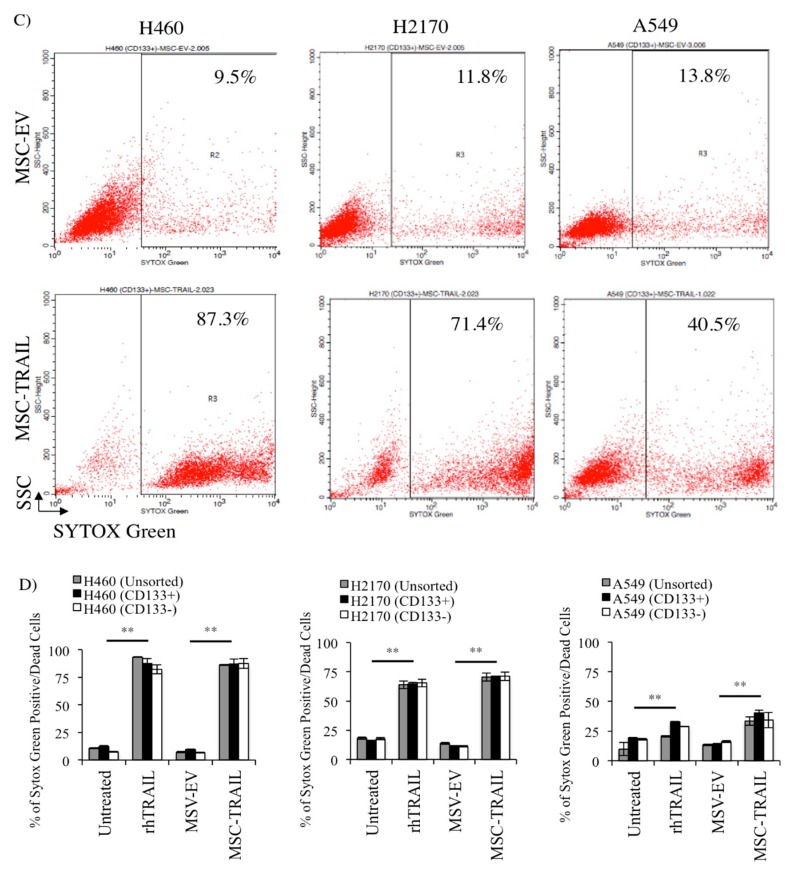
In vitro apoptosis and cell death of CD133+ CSCs by MSC-TRAIL. (**A**) Histograms illustrating the percentage of annexin V expression in CD133+ NSCLC-derived CSCs co-cultured with either MSC-EV or MSC-TRAIL (NSCLC-cell-to-MSC ratio of 1:1) for 72 h, and (**B**) bar chart depicting the percentage of annexin V in CD133+ CSCs and controls (CD133− and unsorted). (**C**) Flow cytometry gating to analyze the percentage of cell death using the Sytox Green assay in CD133+ NSCLC-derived CSCs and controls (CD133− and unsorted) co-cultured with either MSC-EV or MSC-TRAIL. (**D**) MSC-TRAIL significantly induced cell death in the CD133+ CSCs isolated from H469 and H2170, equally as prominent as the effect by rhTRAIL treatment, when compared to the controls (MSC-EV and untreated) (** *p* < 0.001; *t*-test).

To evaluate the efficacy of MSC-TRAIL in inducing annexin V expression (an indicator of apoptosis and cell death) in CD133+ CSCs, both MSC-EV and MSC-TRAIL were co-cultured with the sorted (CD133+ and CD133−) and unsorted NSCLC cell lines (H460, H2170, and A549) at an NSCLC-cell-to-MSC ratio of 1:1 for 72 h (Figure 6). An increase in the percentage of apoptosis by annexin V expression in H460- and H2170-derived CD133+ CSCs treated with MSC-TRAIL (43.3 ± 1.2% and 63.2 ± 1.6%, respectively) as compared to MSC-EV (1.2 ± 0.2% and 4.2 ± 1.3%, respectively) was observed (Figure 6A,B). Moreover, a significantly higher percentage of apoptosis (*p* < 0.001) was also noted in CD133− and unsorted cells derived from H460 and H2170 co-cultured with MSC-TRAIL than in the control (MSC-EV) culture, suggesting that both NSCLC cell lines were highly sensitive to TRAIL-mediated killing (Figure 6B). Furthermore, the apoptotic effect induced by MSC-TRAIL was even higher than the rhTRAIL in both NSCLC cell lines. However, no difference was noted in the percentage of annexin V between the treatments (MSC-TRAIL and rhTRAIL) and controls (untreated and MSC-EV) in the sorted and unsorted A549 cell line.

A substantially higher (*p* < 0.001) percentage of Sytox-Green-positive dead cells in the CD133+ population of H460, H2170, and A549 cultured with MSC-TRAIL (87.3 ± 0.3%, 71.4 ± 0.1%, and 40.5 ± 1.9%, respectively) than that with MSC-EV (9.5 ± 0.4%, 11.8 ± 0.1%, and 13.8 ± 0.7%, respectively) was seen (Figure 6C,D). Moreover, the effect of MSC-TRAIL was equally prominent as the rhTRAIL treatment when compared to the controls (untreated and MSC-EV) (Figure 6D).

### 2.8. MSC-TRAIL Induced Intrinsic Apoptosis in CD133+ NSCLC-Derived CSCs

**Figure 7:**
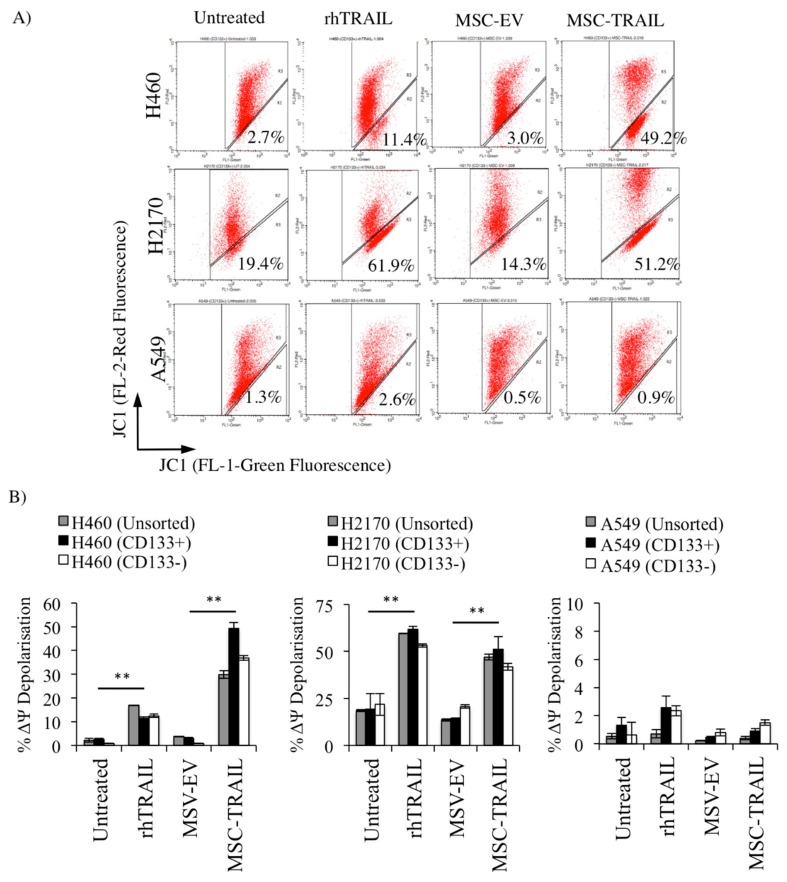
MSC-TRAIL-induced apoptosis through the intrinsic apoptotic pathway. An increase in the mitochondria membrane potential (ΔΨ) depolarization due to the low mitochondria membrane integrity indicates induction of the intrinsic apoptosis pathway. (**A**) Flow cytometry cytograms showing JC-1 stain of CD133+ CSCs from H460, H2170, and A549 cell lines following treatments (MSC-TRAIL or rhTRAIL) and controls (untreated and MSC-EV) groups. (**B**) Significant increase in the percentage of ΔΨ depolarization in NSCLC cell lines (H460 and H2170)-derived CD133+ cells after treatments (MSC-TRAIL or rhTRAIL) as compared to control (MSC-EV and untreated) (** *p* < 0.001; *t*-test).

Through ligand-mediated receptor activation, both the extrinsic and intrinsic apoptotic pathways can be activated. To investigate whether MSC-TRAIL is capable of inducing intrinsic apoptosis in CD133+ CSCs, both the MSCs (MSC-EV and MSC-TRAIL) and CD133+ NSCLC (H460, H2170 and A549)-derived CSCs, as well as the CD133− and unsorted cells, were co-cultured (NSCLC-cell-to-MSC ratio, 1:1) for 72 h and assayed for mitochondrial membrane potential (ΔΨ) (Figure 7). The percentage of JC-1 (tetraethylbenzimidazolylcarbocyanine iodide) monomer (green fluorescence) detected in the FL-1 channel represents the percentage of ΔΨ depolarization and also the degree of activation in the intrinsic apoptotic pathway. Our investigation indicated that the treatment of MSC-TRAIL significantly enhanced the percentage of ΔΨ depolarization in CD133+ NSCLC-derived CSCs when compared to MSC-EV by 16.4-fold (3.0 ± 0.3% to 49.2 ± 2.5% in H460 cell line) and 3.6-fold (14.3 ± 0.3% to 51.1 ± 6.6% in H2170) (Figure 7A). Furthermore, our findings also indicate that the effect of MSC-TRAIL was higher or similar to rhTRAIL in H460 and H2170 (Figure 7A,B). However, no significant effect on the percentage of ΔΨ depolarization was observed when the A549 cell line was treated with MSC-TRAIL or rhTRAIL.

### 2.9. Specific Apoptotic Molecules Regulated in CD133+ H460-Derived CSCs by MSC-TRAIL

**Figure 8:**
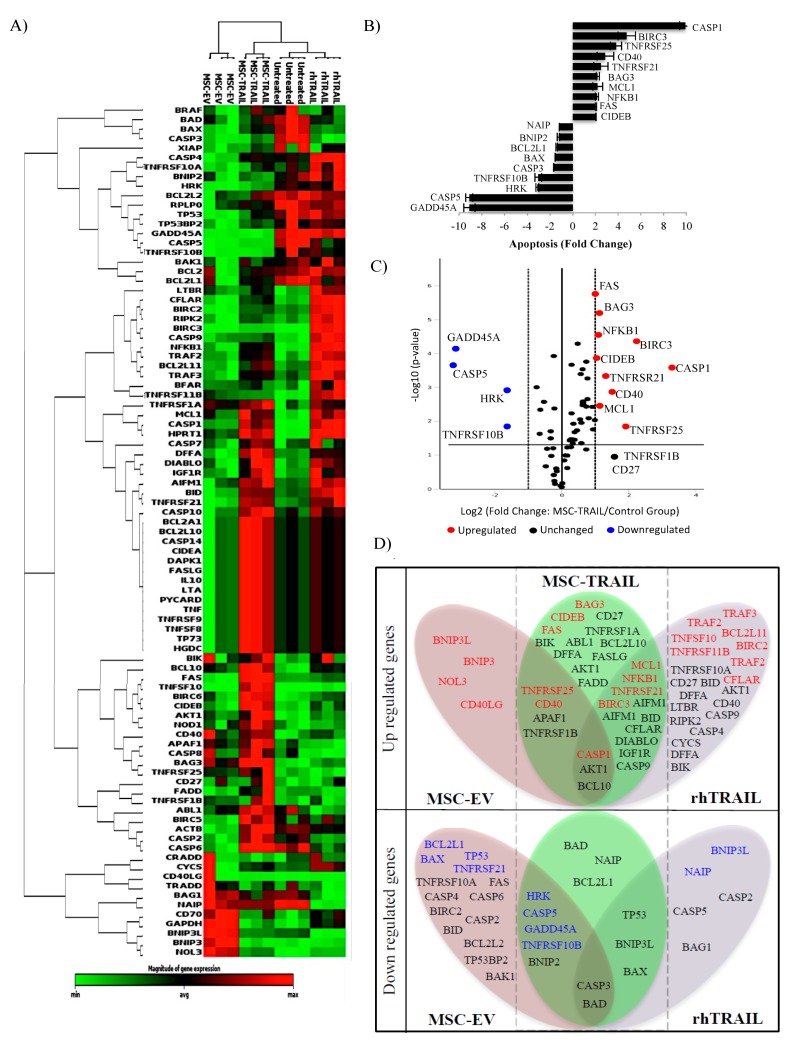
Analysis of apoptotic genes in CD133+ H460-derived CSCs regulated by MSC-TRAIL. (**A**) Hierarchical cluster of CSCs treated with MSC-TRAIL, MSV-EV, rhTRAIL, and untreated control (*n* = 3). (**B**) Bar chart and (**C**) volcano plot showing fold-change (FC) of regulated genes (blue dots/downregulated, FC ≤ 2 and red dots/upregulated, FC ≥ 2.0) with *p* ≤ 0.05 from CD133+ H460-derived CSCs treated with MSC-TRAIL. (**D**) Venn diagram depicting significantly regulated genes (*p* ≤ 0.05) clustered into the three treatment groups (MSC-TRAIL, rhTRAIL, and MSC-EV). The highlighted genes (red and blue) indicate FCs of ≥2 and ≤2, respectively.

We performed pathway-specific gene expression analysis using RT^2^ profiler PCR arrays (Qiagen, Hilden, Germany) to identify apoptotic genes that are uniquely regulated in CD133+ H460-derived CSCs by MSC-TRAIL. The CSCs were subjected to different treatments (MSC-TRAIL, MSC-EV, and rhTRAIL) for 48 h, and untreated CSCs were used as a baseline control (*n* = 3; technical replicates). The gene expression depicted in the heat map between the treatments (MSC-TRAIL and rhTRAIL) and controls (MSC-EV and untreated) was consistent, indicating that the regulation of apoptotic genes by TRAIL was specific (Figure 8A). From all the 84 genes analyzed in the MSC-TRAIL-treated group, 10 genes such as caspase 1 (*CASP1*), baculoviral-encoded inhibitor of apoptosis/IAP repeat containing 3 (*BIRC3*), tumor necrosis factor receptor superfamily member 25 (*TNFRSF25*), cluster of differentiation 40 (*CD40*), *TNFRSF21*, B-cell lymphoma 2/BCL2-associated athanogene 3 (*BAG3*), induced myeloid leukemia cell differentiation protein (*MCL1*), nuclear factor kappa beta subunit 1 (*NFKB1*), fas cell surface death receptor (*FAS*), and cell-death-inducing DNA fragmentation factor-α/DFFA-like effector B (*CIDEB*) were significantly upregulated (*p* ≤ 0.05, fold-change ≥ 2.0; Figure 8B), which are also shown by the red dots in the volcanic plot (Figure 8C). Four genes (growth arrest and DNA damage inducible alpha (*GADD45A*), caspase 5 (*CASP5*), harakiri BCL2-interacting protein (*HRK*), and *TNFRSF10B*) were highly downregulated, as indicated in Figure 8B (*p* ≤ 0.05, fold-change ≤ 2.0), and by the blue dots in Figure 8C. The other five genes (*CASP3*, BCL2-associated X apoptosis regulator (*BAX*), BCL2-like 1 (*BCL2L1*), BCL2-interacting protein 2 (*BNIP2*), and Nod-like receptor/NLR family apoptosis inhibitory protein (*NAIP*)) were downregulated with a fold-change ≤ 1.0 (Figure 8B). A Venn diagram was used to cluster these genes between all the groups (MSC-TRAIL, rhTRAIL and MSC-EV). Among these genes, 14 were similarly regulated (eleven genes upregulated and three genes downregulated) between MSC-TRAIL and rhTRAIL, while nine were modulated (four genes upregulated and five genes downregulated) between the MSC-TRAIL and MSC-EV treatment groups (Figure 8D).

## 3. Discussion

Mesenchymal stem cells as vehicles for anti-tumor cytotherapy are emerging as an alternative approach to the inefficient and highly toxic biological therapies that are currently available. Several sources of mesenchymal stem cells were identified such as adipose tissue, bone marrow, and dental pulp [82,83,84]. However, MSCs harvested from adipose tissues seem more promising considering their high proliferative capacity, ease to obtain, and lack of MHC class II, allowing allogeneic and autologous transplantation without the need for immune suppression. The efficacy of MSC-TRAIL in targeting CSCs is not well studied, and only one report indicated the capacity of MSCs expressing TRAIL to inhibit putative CSCs known as the side population (SP) in NSCLC model [50]. Another study proved the effect of MSCs expressing TRAIL against CD133+ primary glioma cells in an in vitro model [85]; however, this was not in the context of NSCLC. In the present study, we demonstrated for the first time the efficacy of mesenchymal stem cells expressing TRAIL (MSC-TRAIL) against specific CD133+ CSCs isolated from NSCLC cell lines.

Although reports showed that MSCs can be genetically engineered by several gene delivery methods, lentivirus remains as the preferred choice of transduction [86] due to stable transgene expression [87] and better engraftment of transduced cells in the pre-clinical model [88]. Here, we showed that MSCs are malleable to genetic engineering, and that the expression of TRAIL in MSCs through lentivirus transduction was plausible. TRAIL secretion was substantially detected in MSC-TRAIL, and it did not affect cellular viability. Furthermore, MSC-TRAIL maintained its mesodermal characteristics and retained the expression of MSC markers (CD44, CD90, CD105, and CD73). In line with a previous report, our findings also indicated that TRAIL was detected at a very low level from MSCs and MSC-EV [89]. However, our results showed that the natural production of TRAIL by MSC-EV was not effective in inducing a significant anti-tumor effect and, only through exogenous expression of TRAIL from MSC-TRAIL, were the cells capable of inducing a meaningful NSCLC cell inhibition.

A number of studies showed that chemoresistance and tumor recurrence are due to the existence of CSCs that are spared after the initial treatment. Chemotherapies that only target specific pathways or molecules, which are crucial for the survival of non-CSCs, may not be effective in destroying CSCs as they are genetically programmed to be resilient to the common treatments [90,91]. Molecules, surface markers, and receptors that are generally high in CSCs might be ideal candidates to be targeted, as these molecules may be the key regulators of CSC survival [92]. Our results imply that TRAIL cognate receptor (DR5) might contribute toward TRAIL sensitivity in CD133+ CSCs of both H460 and H2170 cell lines. However, we postulate that the contribution of DR4 expression to TRAIL sensitivity in CD133+ CSCs could be observed by increasing the number of cell lines used for the study. Therefore, further analysis looking into the expression of DR4 and other decoy receptors (DcR1 and DcR2) in the CD133+ CSCs is needed to confirm such a finding. Furthermore, compared to NSCLC cell lines, normal cells such as MSCs are generally resistant to TRAIL due to the low cognate receptor DR4 [93] and DR5 expression as depicted in our findings (Figure 2B,C) [45]. Other factors such as the activation of the nuclear factor-kappa beta (NF-κB) [94] and the high expression of the TRAIL decoy receptors may also contribute toward TRAIL resistance in normal cells [95]. This observation further elucidates the specificity of TRAIL and MSC-TRAIL to target and destroy tumors without having an effect on normal cells. Analysis in other non-cancerous cells, such as normal lung epithelial cells, can be made to further confirm the specificity and safety of the TRAIL and MSC-TRAIL.

Even though our finding has indicated low expression of DR4 in H460 and H2170 cell lines, the significance of DR4 must not be understated as it has also been shown to induce apoptosis and was suggested as a potential target for anti-cancer therapy [96]. Furthermore, studies have also shown that both DR4 and DR5 were detected high in lung and liver cancer derived CSCs, signifying their contribution to CSCs TRAIL sensitivity [97,98]. Nevertheless, the function of DR4 and DR5 during TRAIL activation is quite distinct. Glycosylation defects in *N*- and *O*-glycosylation sites of DR4 and DR5, respectively, may alter their function and reduce the sensitivity of tumour cells to TRAIL [99]. For example, mutation in the post-translational modification of DR4 (Serine-424 mutation) has been linked to TRAIL resistance in several cancer cell lines [100]. Furthermore, in contrast to DR5, altering the *N*-glycosylation site in DR4 could also lead to a reduction in overall sensitivity of the cancer cells and CSCs to TRAIL by decreasing TRAIL receptor aggregation and DISC (death inducing signaling complex) formation [101].

Several CSC markers were identified including ALDH [102], ATP-binding cassette super-family G member 2 (ABCG2) [103], CD44 [104], CD326 [105], CD166 [106], and CD133 [107]. However, CD133 is the only marker that is well characterized in many tumors including NSCLC [108,109,110,111]. Current reports utilizing molecular characterization also strengthened the role of the CD133+ population as the CSCs that contribute toward tumorigenesis and chemoresistance in NSCLC [112,113,114]. Furthermore, a meta-analysis in large clinical samples concluded that the high expression of the CD133 marker is strongly correlated with poor overall survival in NSCLC patients [115]. A study also confirmed the presence of CD133+ CSCs in a blood sample of lung cancer patients that further verified the population as the reason for metastasis in lung cancer patients [116].

In the current report, we showed that the CD133+ population isolated from both H460 and H2170 cell lines exhibits the characteristics of CSCs, including bigger and a higher number of tumor spheres and greater clonogenicity (Figure 4A,B). These reflect its tumorigenicity, self-renewal capacity, and high proliferation characteristics [117]. However, the CD133+ population isolated from the A549 cell line may have the characteristics of dormancy or slower proliferation rate, as indicated by the similar sphere size and number in the CD133+ CSCs and control populations (CD133− and unsorted) (Figure 4A). Higher ALDH expression was also detected in all of the CD133+ NSCLC-derived CSCs (Figure 4C) as compared to the CD133− (non-CSCs), suggesting that the population may have greater chemoresistant characteristics [118]. This chemoresistance characteristic of CD133+ can be confirmed by evaluating the cell viability and proliferation. Highest ALDH activity was detected in unsorted A549, suggesting that the heterogeneous population of the unsorted A549 cell line that also consisted of CSCs may have contributed to this observation (Figure 4C).

In this work, we demonstrated that MSC-TRAIL induced tumor cell inhibition, apoptosis, and cell death to the CD133+ CSCs of both H460 and H2170 cell lines. The finding is in line with a previous study that showed the ability of MSC-TRAIL to target the side population (SP) as a model of CSCs in a squamous cell carcinoma cell line [50]. The study also showed that the A549-derived SP was highly sensitive to MSC-TRAIL, unlike our finding that indicates that the A549-derived CD133+ population was quite resistant to both TRAIL and MSC-TRAIL. However, many studies reported that the SP might not be a bona fide CSC. For example, one study reported that the SP was unable to form spheres in an anchorage-independent culture [119]. Another study showed that the non-SP population retained CSCs properties in a pre-clinical model [120], and that the SP population lost its CSCs characteristics and chemoresistance after long-term passaging [121]. We also noted that the CD133− and unsorted cells of both H2170 and H460 cell lines were inhibited by the MSC-TRAIL and rhTRAIL treatments (Figure 5 and Figure 6). This observation may be due to the generally high DR5 receptor expression in the H460 and H2170 cell lines that contributed to the sensitive characteristic of both CD133− and unsorted cells to MSC-TRAIL and rhTRAIL treatments. Moreover, the high expression of DR5 in the CD133− cells of H460 may also contribute to the overall sensitivity of the tumor to TRAIL and MSC-TRAIL. Our findings also indicated that the treatments of MSC-TRAIL and rhTRAIL generated moderate Sytox-Green-positive dead cells in the A549-derived CD133+ CSCs (Figure 6C,D), despite no significant differences in the induction of apoptosis (annexin V expression) and mitochondria membrane potential (ΔΨ) depolarization between the treatment (MSC-TRAIL and rhTRAIL) and control (MSC-EV and untreated) groups. This finding implies that the combination of a DNA dye (such as Sytox Green) and flow cytometry analysis is more sensitive than the luminescence viability assay (Figure 5B) for the detection of dead cells. These results also suggest that the A549-derived CD133+ CSCs might have partial sensitivity to TRAIL and MSC-TRAIL treatments.

It is known that TRAIL and MSC-TRAIL induce an extrinsic apoptosis process through ligand-mediated activation [122,123]. However, the ability of MSC-TRAIL to activate the intrinsic apoptosis, also known as the mitochondria activation of apoptosis, is not reported. We found that MSC-TRAIL was capable of inducing intrinsic apoptosis through mitochondria membrane potential (ΔΨ) depolarization in the CD133+ CSCs of both H460 and H2170 cell lines (Figure 7). Activation of both extrinsic and intrinsic apoptosis through extracellular activation by MSC-TRAIL may induce greater effects, especially for intrinsic-pathway-resistant CSCs that are mostly chemoresistant. This observation is particularly beneficial for cisplatin-resistant CD133+ CSCs [124], whereby sole activation of intrinsic apoptosis by cisplatin treatment may not work to destroy the tumor [125]. We also noted that the A549 cell line was resistant to both intrinsic and extrinsic apoptosis from MSC-TRAIL treatment, which could be related to the low DR5 expression. However, regulatory assays such as western blot looking into specific proteins that contribute toward TRAIL resistance and functional receptor analysis by upregulating DR5 expression in A549 or knockdown experiment in H2170 and H460 cell lines can be done to further confirm such observations. Based on this observation, we believe that, by regulating particular genes in CSCs that contribute toward TRAIL resistance, the effect of MSC-TRAIL on the tumor may be enhanced.

The CD133+ population derived from H460 was selected for gene expression analysis using the RT^2^ profiler PCR arrays due to its intermediate sensitivity to rhTRAIL, unlike A549 and H2170, which were either highly resistant or sensitive to the TRAIL treatment, respectively (Figure 2A). Results from the arrays revealed that treatment of MSC-TRAIL upregulated the expression of 10 apoptosis genes, *CASP1*, *BIRC3*, *TNFRSF25*, *CD40*, *TNFRSF21*, *BAG3*, *MCL1*, *NFKB1*, *FAS*, and *CIDEB* in CD133+ CSCs. In response to the activation of apoptosis by MSC-TRAIL, an increase in mediators of apoptosis, such as *CASP1* and anti-apoptotic effector *BIRC3*, may regulate not only the caspase and apoptosis pathways, but also TRAIL alternative signaling through immunity and inflammatory molecules such as the NF-κB1 [126] and JNK (c-Jun N-terminal kinase) [127]. Upregulation of both *TNFRSF25* and *TNFRSF21* apoptotic regulatory molecules may also have a positive regulation toward an increase in *NFKB1* expression as shown from the results [128]. An increase of *NFKB1* in CD133+ CSCs during MSC-TRAIL activation may pinpoint specific signaling cascades important for maintaining the characteristics of CSCs, such as the capacity for self-renewal, migration, invasion, and chemoresistance particularly toward TRAIL and MSC-TRAIL therapy [129]. Furthermore, the inhibition of *NFKB1* activity using proteasome inhibitor (MG132) was reported to enhance TRAIL-mediated apoptosis in NSCLC cell lines (A549 and NCI-H1299) [130]. Therefore, we postulate that a pre-treatment targeting *NFKB1* could increase the efficacy of TRAIL and MSC-TRAIL-mediated inhibition through CD133+ CSC sensitization in NSCLC. Moreover, upregulation of both *BAG3* and *MCL1* may have a negative effect toward MSC-TRAIL and apoptosis induction [131,132], and knocking down both genes through pre-treatment in NSCLC may sensitize the CD133+ CSCs to the effect of MSC-TRAIL-mediated inhibition. The treatment of MSC-TRAIL did not have an effect toward an increase in *GADD45A* expression in the CD133+ CSC sample corresponding to a report that showed low expression of *GADD45A* in NSCLC tissue samples [133]. However, upregulating the *GADD45A* gene using histone deacetylase (HDAC) inhibitor [134] may enhance the effect of TRAIL and MSC-TRAIL in NSCLC through negative regulation in *NFKB1* expression and the inhibition of CSCs [135,136]. Our findings also indicated that the treatment of MSC-TRAIL downregulated the expression of the *HRK* gene in the CD133+ CSCs. Based on this observation and from a previous report that showed the role of HRK in apoptosis, the overexpression of the *HRK* gene in the CD133+ CSCs might enhance the effect of MSC-TRAIL in NSCLC through CSC sensitization [137].

## 4. Materials and Methods

### 4.1. Cell Culture

Human adipose-derived mesenchymal stem cells (MSCs; cat no: ATCC^®^ PCS-500-011) were purchased from the American Type Culture Collection (ATCC, Manassas, VA, USA). The MSCs were cultured in specific growth medium containing knockout Dulbecco’s Modified Eagle Medium (DMEM), 1% penicillin/streptomycin, 2 mM l-glutamine (200 mM stock), 10% fetal bovine serum (FBS), 5 ng/mL fibroblast growth factor (FGF) basic, and 5 ng/mL recombinant epidermal growth factor (rhEGF). The human embryonic kidney cell line (Lenti-X 293T, cat no: 632180) was purchased from Takara Bio Inc, (Takara Bio, Clontech, Mountain View, CA, USA), cultured in DMEM with high glucose (4.5 g/L), 4 mM l-glutamine, sodium pyruvate supplemented with 10% FBS, and 1% penicillin/streptomycin.

Three types of human non-small cell lung cancer cell lines (H2170, A549, and H460) were used in this study. Lung squamous cell carcinoma (H2170) (cat no: ATCC^®^ CRL-5928) was purchased from the American Type Culture Collection (ATCC, Manassas, VA, USA), while the other two cell lines (constitutively expressed luciferase), human adenocarcinoma (A549) (cat no: JCRB1414) and large cell lung cancer (H460) (cat no: JCRB1407), were purchased from Cell Bank Australia (Westmead, NSW, Australia). The A549 cells were grown in Roswell Park Memorial Institute-1640 (RPMI)-1640 complete medium containing 1% penicillin/streptomycin, 1× non-essential amino-acid solution, and 10% heat-inactivated FBS. For the H460 cell line, complete medium was prepared by adding 15% heat-inactivated FBS to RPMI-1640 medium containing 1% penicillin/streptomycin and 0.8 μg/mL insulin (4 mg/mL stock). H2170 complete medium was prepared by adding 10% FBS and 1% penicillin/streptomycin to RPMI-1640.

The cells were maintained in 75-cm^2^ flasks (Nunc, Thermo Fisher Scientific, Inc., Waltham, MA, USA) and harvested using 0.25% trypsin–ethylenediaminetetraacetic acid (EDTA) when cells reached 80% confluence. All cells were grown at 37 °C in a humidified atmosphere of 5% CO_2_. All culture reagents were obtained from Gibco (Thermo Fisher Scientific, Inc., Waltham, MA, USA).

### 4.2. Lentivirus Production and MSC Transduction

The human full-length TRAIL gene (NM_003810.2) was amplified from complementary DNA (cDNA) derived from MSCs using CloneAmp HiFi PCR Premix (Takara). Total RNA from MSCs was isolated using the RNeasy mini kit and the cDNA was synthesized using the QuantiTect Reverse Transcription Kit according to the manufacturer’s recommendations (Qiagen, Hilden, Germany).

Both the primers (5′–TATTTCCGGTGAATTATGGCTATGATGGAGGTCC–3′ (forward), 5′–GAGAGGGGCGGGATCTTAGCCAACTAAAAAGGCCC–3′ (reverse)) used for the cDNA amplification contained 15-bp overlaps at their ends that allowed complementary base pairs between the synthesized TRAIL–cDNA and the bicistronic lentiviral expression vector (pLVX-EF1α-mCherry). Ligation between TRAIL–cDNA and the expression vector was performed through homologous end-joining using an in-fusion HD cloning kit (Takara Bio, Clontech, Mountain View, CA, USA) according to the manufacturer’s recommendations. The resulting vectors were termed as pLVX-EF1α-TRAIL-mCherry and pLVX-EF1α-empty vector (EV)-mCherry as the control. The lentivirus was produced by transfecting Lenti-X 293T (2.0 × 10^6^ in a 10-mm petri dish) with a solution containing 7 μg of transfer plasmid (pLVX-EF1α-TRAIL-mCherry or pLVX-EF1α-EV-mCherry), packaging plasmids (3.5 μg of pCMV-VSV-G and 7 μg of psPAX2 (Addgene, Watertown, MA, USA), 50 μL of Fugene HD (Roche, Basel, Switzerland), and DMEM (added to 600 μL). The lentiviruses produced (TRAIL-encoding lentivirus and empty vector/EV-encoding lentivirus) were concentrated using Lenti-X concentrator (Takara), aliquot and stored at −80 °C until further use. Concentrated lentiviruses resulted in >10^7^ transducing units (TU)/mL, and the mean multiplicity of infection (MOI) (calculated from the TU) of 20 and 10 μg/mL polybrene was used to transduce the MSCs.

### 4.3. Characterizations of MSC-TRAIL

The transduced MSCs (MSCs transduced with TRAIL-encoding lentivirus (MSC-TRAIL) and MSCs transduced with empty (EV)-encoding lentivirus (MSC-EV)) expressed mCherry were quantified using flow cytometry. Briefly, six days after transduction, cells were detached using 0.25% trypsin–EDTA and pelleted by centrifugation, before being suspended in Dulbecco’s phosphate-buffered saline (DPBS) containing 2% FBS. The percentage of transduction efficiency (mCherry+) was analyzed by acquiring 10,000 events analyzed using FACS Calibur (Becton Dickinson BD, NJ, USA) gated on the FL-3 channel. MSC-TRAIL was also stained with specific MSC antibodies (CD44-PE, CD90-PE, CD105-PE, and CD73-PE) and subsequently analyzed using flow cytometry, gated on mCherry-negative cells and the FL-2 channel. The human TRAIL expression by MSC-TRAIL was validated using ELISA (RayBiotech, Norcross, GA, USA). MSC-TRAIL was induced into the mesodermal lineage by culturing in adipogenesis medium for seven days, osteogenesis medium for 21 days, and chondrogenesis medium for 14 days. The differentiated cells were subsequently stained with Oil-Red O, Alizarin Red S, and Alcian Blue, respectively. The differentiation process was performed following the protocol recommended by the manufacturer (Gibco, Thermo Fisher Scientific, Inc., Waltham, MA, USA).

### 4.4. Isolation of CD133+ NSCLC-Derived CSCs

The NSCLC cell lines (A549, H460 and H2170) were harvested by 5-min incubation with 0.25% trypsin–EDTA followed by centrifugation. The cell pellets were then suspended in DPBS with 2% FBS and transferred into 75-mm polystyrene round-bottom test tubes. CD133-PE antibody, 1:10 dilution (Clone: AC133; Isotype: Mouse IgG1 _kappa_) (Miltenyi Biotec, Bergisch Gladbach, Germany), was added to the cell suspension and incubated for 15 min in dark. Cells were then washed with DPBS containing 2% FBS and filtered through a 40-μM cell strainer, before being subjected to specific CD133+ (cancer stem cells, CSCs) and CD133− (non-CSCs) isolation using a fluorescence-activated cell sorter (FACSAria III; BD biosciences, San Jose, CA, USA). The isolated CD133+ CSCs were grown in complete RPMI-1640 medium and supplemented according to cell types (described as complete medium for H460, H2170, and A549), and re-sorted for another two cycles for a total of three sorting experiments to get a pure CD133+ population.

### 4.5. TRAIL Agonist (DR4 and DR5) and Decoy (DcR1 and DcR2) Receptors Expression in NSCLC Cell Lines

The NSCLC cell lines were analyzed for TRAIL agonist (DR4 and DR5) and other decoy receptors (DcR1 and DcR2) with allophycocyanin (APC)-conjugated TRAIL-R1 (DR4) (cat no: FAB347A; Mouse IgG1), TRAIL-R2 (DR5) (cat no: FAB6311A; Mouse IgG_2B_), TRAIL-R3 (DcR1) (cat no: FAB6302A; Mouse IgG1), and TRAIL-R4 (DcR2) (cat no: FAB633A; Mouse IgG1) (R&D Systems, Minneapolis, MN, USA) using flow cytometry (Becton Dickinson BD). Briefly, NSCLC cells (1.0 × 10^6^ cells in 200 μL of DPBS) were stained with the antibodies (5 mL of CD133-PE and DR5-APC) for 30 min on ice in the dark. After the washing steps, stained cells were subjected to flow cytometric analysis using the FACS Calibur instrument (Becton Dickinson BD), and a total of 10,000 events were acquired and analyzed using Cell Quest software (Becton Dickinson BD).

### 4.6. CSC Characterization (Sphere Formation and Clonogenic Assays)

The sorted (CD133+ and CD133−) and unsorted populations were cultured in 24-well ultra-low attachment plates (1.0 × 10^3^ cells/mL) in serum-free medium containing RPMI-1640, 10 ng/mL fibroblast growth factor (bFGF), 1% B27, 20 ng/mL EGF, and 1% penicillin/streptomycin (Gibco, Thermo Fisher Scientific, Inc., Waltham, MA, USA). Cells were cultured until spheres could be visually detected and analyzed (minimum of 20 fields at 100× magnification) using CK40 light microscopy (Olympus, Waltham, MA, USA). For the clonogenic assay, NSCLC cell lines (sorted and unsorted) were grown in complete medium (500 cells/mL) in a six-well plate for 14 days and subsequently fixed with 4% paraformaldehyde for 10 min, before being stained using crystal violet for 30 min and washed using DPBS. The number of colonies formed was counted manually for three independent experiments.

### 4.7. Aldehyde Dehydrogenase (ALDH) Activity (Aldefluor Assay)

The analysis of ALDH activity in sorted and unsorted NSCLC cell lines was performed according to the manufacturer’s recommendations (StemCell Technologies, Vancouver, BC, Canada). Briefly, the sorted and unsorted NSCLC cells (1.0 × 10^6^ cells) were collected by centrifugation and suspended in 1 mL of assay buffer. Subsequently, samples were divided into two tubes labeled as test and control containing 5.0 × 10^5^ cells in 500 μL of assay buffer. ALDH inhibitor (*N*,*N*-diethylaminobenzaldehyde/DEAB; 5 μL) was added to the control tube, followed by Aldefluor stain (2.5 μL) into the test tube. Immediately after the addition of Aldefluor stain to the test tube, 500 μL of the test sample was added into the control tube and incubated for 30 min in the dark. Cells were subsequently washed, pelleted by centrifugation, re-suspended in 500 µL of assay buffer, subjected to flow cytometry (BD FACS Calibur; BD biosciences), and analyzed using BD Cellquest Pro software.

### 4.8. Cell Proliferation/MTS Assay

MTS solution (CellTiter 96^®^ Aqueous One Solution Cell Proliferation Assay; Promega Corporation, Madison, WI, USA) was used to determine the IC_50_ value of the NSCLC cell lines (A549, H2170, and H460) to recombinant human TRAIL (rhTRAIL), and to define resistant and sensitive cell lines based on cell viability after rhTRAIL (500 ng/mL) treatment. The cells were seeded in a 96-well plate (1.0 × 10^4^ cells/well) overnight. The rhTRAIL (BioVision, Milpitas, CA, USA) at different concentrations (0.8–12.5 µg/mL) was added to the cell lines for 48 h in a humidified 5% CO_2_ incubator at 37 °C. Following incubation, 20 μL of MTS solution was added into the wells and incubated for 4 h. After the incubation period, absorbance was assessed using an Envision 2103-0020 plate reader at 490 nm (Perkin Elmer, Waltham, MA, USA). Cell proliferation was calculated according to the following formula: cell proliferation (%) = (absorbance (cells with treatment)/absorbance (cells without treatment)) × 100. The IC_50_ value of rhTRAIL in each of the cell lines was calculated using a linear regression formula (*y* = m*x* + c) from a scatter plot, whereby the *x*-axis was the concentration of TRAIL (in log10), *y* was the percentage of cell viability, m was the gradient, and c was the *y*-intercept value. The IC_50_ value of TRAIL was then calculated by anti-log of the derived TRAIL concentration value (in log10) from 50% of cell viability [138,139].

### 4.9. Luciferase Assay

Since both A549 and H460 constitutively produce luciferase, the viability of these cells after treatment was reflected by the activity of luciferase to convert luciferin into oxyluciferin, which can be detected as luminescence. Briefly, sorted (CD133+ and CD133−) and unsorted A549 and H460 cells were initially seeded in a 96-well tissue culture plate at 1.0 × 10^3^ cells/well in complete medium. Then, MSC-TRAIL and MSC-EV were added according to different NSCLC-cell-to-MSC ratios (1:1, 1:3, and 1:6). Treatment with rhTRAIL according to the IC_50_ value of each cell (12.6 ng/mL for H2170, 218 ng/mL for H2170, and 500 ng/mL for A549) was used as a positive control for the experiment. After 72 h, d-luciferin (150 μg/mL) was added to each well, and the plate was subjected to luminescence reading at 580 nm using an Envision 2103-0020 plate reader (Perkin Elmer, Waltham, MA, USA). Luminescence (fold-change) of both sorted and unsorted A549 and H460 cell lines was calculated as luminescence value (with treatments)/luminescence value (untreated). For analysis of luminescence in H2170 (this cell line does not express luciferase), CellTitre-Glo (Promega) was used and the same protocol was applied. However, modification in the analysis of fold-change (luminescence) for H2170 was made as follows: [(luminescence value H2170 (with treatment)) − luminescence value of MSC-EV or MSC-TRAIL)]/luminescence value of H2170 (untreated).

### 4.10. Apoptosis and Dead Cell Analysis

Analysis of apoptosis was evaluated using FITC-conjugated annexin V antibody from BD Pharmingen (Becton Dickinson Biosciences, San Jose, CA, USA). In brief, sorted and unsorted NSCLC cell lines (A549, H460, and H2170) were initially seeded (5.0 × 10^4^ in 200 μL of medium) in a 24-well tissue culture plate. Next, treatments of MSC-EV, MSC-TRAIL (5.0 × 10^4^ in 200 μL of medium), or rhTRAIL (IC_50_ value of 12.6 ng/mL for H2170, 218 ng/mL for H2170, and 500 ng/mL for A549) were added to the wells. After 72 h, cells were harvested by trypsinization and collected by centrifugation. Annexin V binding buffer (100 μL) was added with 1 μL of annexin V–FITC antibody and incubated at 4 °C in the dark. For dead cell analysis, 1 μL of Sytox Green (Invitrogen, Thermo Fisher Scientific, Inc., Waltham, MA, USA) was added to 500 μL of cell suspension and directly used for analysis. Stained cells were subjected to flow cytometric analysis, and 10,000 events were collected using the FACS Calibur instrument (Becton Dickinson BD) and analyzed using Cell Quest Pro software gated on FL-1 (Becton Dickinson BD).

### 4.11. Mitochondria Membrane Potential (ΔΨ)

To look into the activation of the intrinsic apoptosis in sorted and unsorted NSCLC cell lines post-treatment, we conducted mitochondria membrane potential (ΔΨ) analysis using the BD MitoScreen (Becton Dickinson Biosciences, San Jose, CA, USA). Sorted and unsorted NSCLC cell lines were seeded with MSC-TRAIL, MSC-EV at 1:1 ratio, or rhTRAIL (IC_50_ value of 12.6 ng/mL for H2170, 218 ng/mL for H2170, and 500 ng/mL for A549) into a 24-well tissue culture plate. After 72 h of incubation, cells were trypsinized and collected by centrifugation, before being suspended in 250 μL of JC-1 stain (diluted from stock) and incubated for 15 min in the dark. Stained cells were then washed twice with assay buffer and pelleted by centrifugation. Collected cells were suspended in DPBS containing 2% FBS and kept on ice. Analysis was performed using the FACS Calibur instrument (Becton Dickinson BD) and gated at FL-1 (green fluorescence) and FL-2 (red fluorescence) channel.

### 4.12. RT^2^ Profiler PCR Array

The 84 key genes and biological pathways involved in cell death activation by TRAIL were analyzed in CD133+ H460-derived CSCs using the Apoptosis RT^2^ Profiler PCR Array (PAHS-012Z; Qiagen, MD, USA). The human apoptosis RT² profiler PCR array profiles the transcriptional level using quantitative RT-PCR of 84 critical genes categorically divided into anti-apoptosis, regulators of apoptosis, death domain receptors, and caspases. Sorted and unsorted NSCLC cell lines were seeded with MSC-TRAIL, MSC-EV (1:1 ratio), or rhTRAIL (IC_50_ value) in a 24-well tissue culture plate. After 48 h, total RNA was extracted from samples and on-column DNA digestion was performed during the extraction process. RNA (0.5 μg) was added into the genomic DNA elimination mix to a total of 10 μL. The sample was incubated at 42 °C for 5 min and then on ice for 1 min. Reverse transcription mix (10 μL) was then added, and the sample was incubated at 42 °C for 15 min and 95 °C for 5 min. After the reaction time, 91 μL of RNAse-free water was added to make a total of 111 μL of cDNA synthesizing reaction. The PCR component mix was prepared by adding 102 μL of cDNA synthesizing reaction to 2× RT^2^ SYBR Green Mastermix and RNase-free water to a total volume of 2700 μL. Subsequently, 25 μL of the reaction was pipetted into each well of the 96-well PCR array plate and centrifuged at room temperature for 1 min at 1000× *g* (all reagents were purchased from Qiagen, MD, USA). The PCR cycling process (one cycle at 95 °C for 10 min, followed by 40 cycles of 95 °C for 15 s and 60 °C for 60 s) was performed using LightCycler 480 (Roche, Indianapolis, IN, USA). Analyses of data were performed using web-based analysis software [140].

### 4.13. Statistical Analysis

Data are presented as means ± standard deviation (SD) of three independent experiments. Comparison between two groups was performed using the two-tailed *t*-test with *p* < 0.05 considered statistically significant. Comparison between groups was performed using one-factor analysis of variance (ANOVA). Analyses were performed using Excel 2010, version 14.0 (Microsoft Corporation, Redmond, WA, USA).

## 5. Conclusions

Collectively, our results revealed that NSCLC-derived CD133+ CSCs can be effectively targeted using MSC-TRAIL through the extrinsic and intrinsic apoptosis pathways. While common chemotherapy may not be effective in destroying CSCs and tumor cells in general, MSC-TRAIL could serve as a better alternative or as a complement to the treatment in NSCLC patients. Finally, through gene expression analysis, we identified signaling molecules that might contribute toward TRAIL and MSC-TRAIL resistance in CD133+ CSCs, and uncovered *NFKB1*, *BAG3*, *MCL1*, *GADD45A*, and *HRK* as potential anti-cancer genes that could increase sensitivity of NSCLC to MSC-TRAIL therapy. To further understand the mechanism of TRAIL resistance in the CD133+ CSCs, functional assays incorporating MSC-TRAIL and a knockdown or sensitized model of the CSCs and NSCLC cell lines can be performed in the future.
